# A Global Perspective on the Controversy of Gleason Score 6 Prostate Cancer Reporting: The Potential Role of Population-Based Cancer Registries

**DOI:** 10.1200/GO.22.00427

**Published:** 2023-04-26

**Authors:** Xavier Farré, Seyed Mohsen Mousavi, Rafael Marcos-Gragera, Kamal Malaker

**Affiliations:** Xavier Farré, MD, PhD, Department of Health, Public Health Agency of Catalonia, Lleida, Spain; Seyed Mohsen Mousavi, MD, East Switzerland Cancer Registry, St Gallen, Switzerland; Rafael Marcos-Gragera, MD, PhD, Epidemiology Unit and Girona Cancer Registry, Catalan Institute of Oncology, Girona Biomedical Research Institute Dr Josep Trueta, Girona, Spain; and Kamal Malaker, MD, PhD, North Arc Health Consultancy Services, Singapore, Singapore

## TO THE EDITOR:

Issues associated with prostate cancer (PCa) overdiagnosis and overtreatment in high-income countries have reopened the debate on whether Gleason score 6 (GS6) should be labeled as a precursor lesion and no longer be mandatorily diagnosed, treated, or actively monitored.^1-3^

Gleason scoring oversimplifies a disparate number of architectural cancer patterns. At pathology diagnosis, some patterns present enough subjectivity to blur the boundary between GS6 and higher Gleason scores, thereby producing limited overall agreement among pathologists on the reporting of Gleason scores. Furthermore, GS6 on biopsy can be upstaged when cases undergo radical prostatectomy. In addition, PCa from any sample may be restaged after a second opinion by another pathologist. Finally, whenever cases are studied molecularly, genomic alterations and chromosomal abnormalities frequently overlap between GS6 and higher Gleason scores.^4,5^

Most of this evidence comes from studies with data obtained from medical records and research databases, which cannot encompass all features of GS6 PCa. Instead, appropriately equipped and staffed population-based cancer registries offer high-quality standardized data to study GS6 PCa within a defined geographical area around the world.^6^ Furthermore, specialized PCa population-based cancer registries have the potential to collect additional data on risk factors, screening, and therapy.^7^

Most PCa research studies are done on high-income countries, where the management of PCa has evolved to using multiparametric magnetic resonance imaging for targeting biopsies at highly suspicious Prostate Imaging Reporting and Data System (PI-RADS) lesions of PCa, and many patients with low-volume GS6 PCa are increasingly managed conservatively.^8^ In addition, active surveillance has enabled a delay in active treatment over the past two decades, and the rates of GS6 PCa at prostatectomy have fallen. Furthermore, commercial tests used for GS6 stratification help decide the use of conservative treatments. That level of disease management that is easily accessible in high-income countries is not realistic in low-income countries, where late diagnosis of PCa is common, and advances in early diagnosis and management of PCa are needed.^9^

When looking at PCa estimated incidence rate, mortality, and survival data available from low-income countries, we should suspect that PCa biology may be different compared with that from high-income countries. For instance, the Caribbean has areas with the highest incidence and mortality in the world and, at the same time, limited PCa screening, leading to later diagnosis.^10^

Low-income countries face tremendous scarcity of investment and resources. Local health care systems are undersupplied in terms of diagnostic facilities and trained staff. PCa screening is limited, with a lack of early detection services and public awareness. Consequently, PCa is underdiagnosed and detected at late stages presenting with symptomatic disease. PCa is also undertreated as patients may not be able to pay for treatment, and appropriate facilities for advanced cancer treatments may not be available. Thus, population-based registries in low-income countries often record limited information on PCa. For instance, there are low rates of information on PSA levels at diagnosis, Gleason score, TNM stage, imaging techniques, adequate diagnostic follow-up, and treatment received.^9^

Appropriately equipped and staffed population-based cancer registries have the potential to record large volumes of cases in any part of the world. From such data, we could accurately measure the existence of GS6 cases with metastasis and confirm its indolent behavior. Cases with a GS6 biopsy and metastasis are described in large population-based cancer registries from high-income countries that have many years of experience registering cancer data. The problem with these cases is that they are not usually treated with radical prostatectomy, and therefore, it cannot be ruled out that nonbiopsied areas could harbor higher Gleason grade areas. Thus, in the absence of radical prostatectomy, we cannot be certain if the removed PCa is indeed GS6.^7^

In conclusion, there is a controversial debate about GS6, assuming the features of PCa in high-income countries, but without a global study of the disease and without complete data on PCa in low-income countries. In an era of rapid technological advances, we may think that large global networks of population-based cancer registries would be created and give us access to data from any country in the world, without income consideration. In terms of the current knowledge on PCa, we have not yet defined, based on the complete study of the organ, the likely existence of a subset of confirmed GS6 cases that could be potentially lethal and should be safety monitored. In this scenario, GS6 should not be labeled as a precursor lesion or as indolent until further evidence is confirmed by global data.
